# Multiple functions of m^6^A RNA methylation in cancer

**DOI:** 10.1186/s13045-018-0590-8

**Published:** 2018-03-27

**Authors:** Yutian Pan, Pei Ma, Yu Liu, Wei Li, Yongqian Shu

**Affiliations:** 1 Department of Oncology, The First Affiliated Hospital of Nanjing Medical University, Nanjing, People’s Republic of China; 2 Jiangsu Key Lab of Cancer Biomarkers, Prevention and Treatment, Collaborative Innovation Center for Cancer Personalized Medicine, Nanjing Medical University, Nanjing, People’s Republic of China; 3 Department of Orthopaedics, Shanghai General Hospital, Shanghai Jiao Tong University School of Medicine, Shanghai, People’s Republic of China; 4 Department of Oncology, Sir Run Run Hospital, Nanjing Medical University, Nanjing, People’s Republic of China

**Keywords:** m^6^A, RNA methylation, Cancer, Mechanism

## Abstract

First identified in 1974, m^6^A RNA methylation, which serves as a predominant internal modification of RNA in higher eukaryotes, has gained prodigious interest in recent years. Modifications of m^6^A are dynamic and reversible in mammalian cells, which have been proposed as another layer of epigenetic regulation similar to DNA and histone modifications. m^6^A RNA methylation is involved in all stages in the life cycle of RNA, ranging from RNA processing, through nuclear export, translation modulation to RNA degradation, which suggests its potential of influencing a plurality of aspects of RNA metabolism. All of the recent studies have pointed to a complicated regulation network of m^6^A modification in different tissues, cell lines, and space–time models. m^6^A methylation has been found to have an impact on tumor initiation and progression through various mechanisms. Furthermore, m^6^A RNA methylation has provided new opportunities for early stage diagnosis and treatment of cancers.

Herein, we review the chemical basis of m^6^A RNA methylation, its multiple functions and potential significance in cancer.

## Background

Termed as “epitranscriptome,” RNA harbors the potential of being dynamically and reversibly regulated by the addition and removal of distinct chemical moieties, which extends the RNA repertoire and alters its chemistry in various ways [1].

Among more than 100 types of post-transcriptional modifications identified in RNAs so far [2–4], *N*^6^-methyladenosine (m^6^A) RNA methylation is one of the most prevalent modifications, accounting for about 50% of total methylated ribonucleotides and 0.1–0.4% of all adenosines in total cellular RNAs [5, 6]. Most mammalian m^6^A sites are found within the consensus sequence Rm^6^ACH (R = G or A, H = A, C, or U), which is consistent with the enriched binding motifs observed in studies of methyltransferase-like 14 (METTL14), methyltransferase-like 3 (METTL3), and Wilms tumor 1-associated protein (WTAP) (GGAC, GGAC, and GACU respectively) [7]. There have been plenty of researches concerning DNA and histone methylation. DNA methylation is an important biochemical process which comprises the addition of a methyl group to the carbon 5 of the pyrimidine ring of cytosine or the nitrogen 6 of the purine ring of adenine. DNA methylation is brought about by a group of enzymes known as the DNA methyltransferases (DNMT) and can be de novo (when CpG dinucleotides on both DNA strands are unmethylated) or maintenance (when CpG dinucleotides on one strand are methylated). The enzymes that actively demethylate DNA include 5-methylcytosine glycosylase and MBD2b. Accumulating evidence has shown that DNA methylation plays an important role in all aspects of cellular processes including tumorigenesis [8].

Histone methylation in localized promoter regions is one kind of histone codes for chromatin packing and transcription. Generally, methylation of H3K4, H3K36, and H3K79 is linked to gene expression activation, whereas H3K9me2, H3K9me3, H3K27me3, and H4K20 are associated with gene repression [9–12]. Modifications of m^6^A RNA methylation are dynamic and reversible in mammalian cells, which have been proposed as another layer of epigenetic regulation similar to DNA and histone methylation. The biological function of m^6^A RNA methylation is highly variable depending on context and little is known about the underlying mechanisms; however, emerging evidence has suggested that m^6^A modification plays a pivotal role in pre-mRNA splicing, 3′-end processing, nuclear export, translation regulation, mRNA decay, and miRNA processing [13]. Growing appreciation of the biological significance of m^6^A RNA methylation has implied its important and diverse biological functions in mammals including tissue development, circadian rhythm, DNA damage response, sex determination, and tumorigenesis [14].

In this review, we will discuss how m^6^A RNA methylation participates in tumorigenesis, invasion, metastasis, and drug resistance and how to use m^6^A modification as new diagnostic biomarkers and therapeutic targets.

## Chemical basis of m^6^A RNA methylation

The m^6^A methylase complex is consisted of at least five “writer” proteins where METTL3 acts as the catalytic core. METTL14 serves as structural support for METTL3, while WTAP stabilizes the core complex. RNA-binding motif protein 15 (RBM15) helps to recruit the complex to its target sites. The molecular function of KIAA1429 is still elusive [15]. On the other hand, “erasers” consisting of fat mass and obesity-associated protein (FTO) and alkB homolog 5 (ALKBH5) act as demethylases to reverse m^6^A modification [16, 17]. The functional interplay among the m^6^A methyltransferases and demethylases probably determines the dynamic regulation of m^6^A modification.

YT521-B homology (YTH) domain-containing proteins including YTHDF1-3, YTHDC1, and YTHDC2 have recently been identified as “readers” of m^6^A marks on mRNA. They display a 10- to 50-fold higher affinity for m^6^A-methylated mRNA than for unmethylated mRNA [18–22]. When perturbed in different cellular contexts, individual YTH domain family proteins interact with distinct subsets of m^6^A sites and produce different effects on gene expression. For example, YTHDF2, the first identified m^6^A reader, increases turnover of m^6^A-modified mRNA by promoting co-localization with decay factors [23, 24]. Conversely, YTHDF1 can be bound to m^6^A sites near the stop codon, which then interacts with the translation initiation factor eIF3, causing a stimulatory effect on translation in mammals [25]. Another mechanism for the regulation of translation by m^6^A RNA methylation comes into play during stress responses where preferential m^6^A methylation of the 5′-UTR of stress response genes leads to cap-independent translation, which involves nuclear relocalization of YTHDF2 to prevent m^6^A removal by FTO [26, 27]. YTHDF3 has been shown to interact cooperatively with YTHDF2 to accelerate mRNA decay while it also promotes translation of methylated RNAs in cooperation with YTHDF1 [28, 29]. YTHDC1 recruits the splicing factor Serine and arginine-rich splicing factor (SRSF) 3, promotes exon inclusion, and restricts binding of the exon-skipping factor SRSF10 [30].

As to the processing of pri-miRNAs, hnRNPA2B1 has the capability of recruiting DGCR8 to RNA by targeting m^6^A sites, indicating its important role in promoting pri-miRNA processing [31, 32].

A special combination of direct and indirect mediation has also been observed and studied. m^6^A RNA methylation has been shown to modify RNA structure, as a direct effect, in a way that improves accessibility for RNA-binding proteins such as hnRNPC, which in turn mediates an indirect effect. This phenomenon is termed as “m^6^A-switch” [33].

**Fig. 1:**
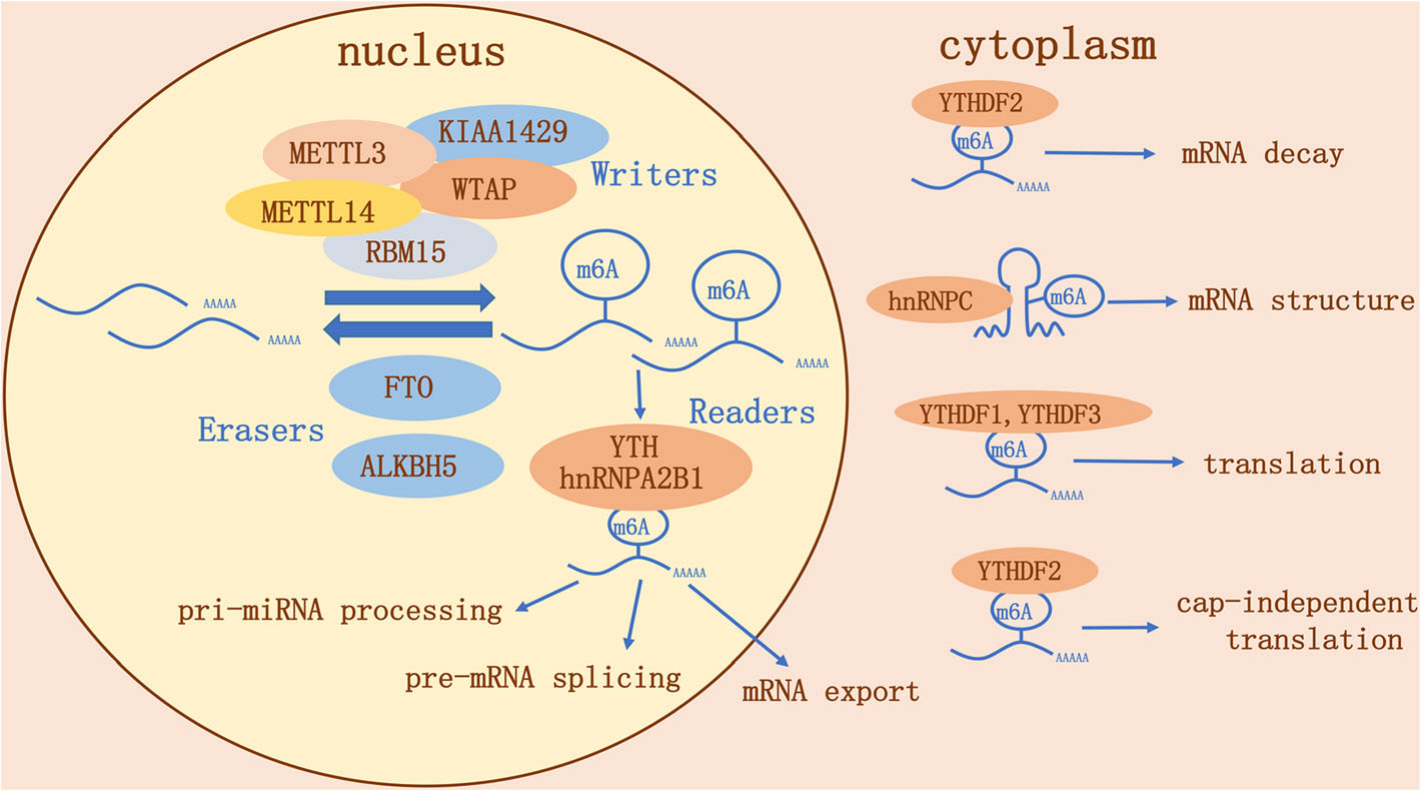
Chemical basis and diverse molecular functions of m^6^A RNA methylation. m^6^A RNA methylation is modulated by its “writers,” “erasers,” and “readers.” Writers refer to the m^6^A methylase complex which is consisted of METTL3, METTL14, WTAP, RBM15, and KIAA1429. Erasers are m^6^A demethylases including FTO and ALKBH5. Readers are proteins that bind to m^6^A modifications and exert various functions, some of whom identified so far are YTHDF1, YTHDF2, YTHDF3, YTHDC1, YTHDC2, and hnRNPA2B1. m^6^A RNA methylation is known to be involved in all stages in the life cycle of RNA including pre-mRNA splicing, pri-miRNA processing, through nuclear export, RNA translation modulation and RNA degradation

To sum up, the emerging new data and discoveries have revealed a complex picture concerning epitranscriptome (Fig. 1).

## Methods of detecting m^6^A RNA methylation

The research related with m^6^A modification has been reinvigorated due to the advent of potent analytical methods, which harness the effects of m^6^A RNA methylation on RNA structure and metabolism, in combination with novel, high-throughput methods.

Several methods, including dot-blot and high-performance liquid chromatography coupled to triple–quadrupole mass spectrometry (LC–MS/MS) can be utilized to yield important quantitative information about existence and abundance of m^6^A modification, while they are not suitable for widespread identification and localization of modified sites [34].

Thus, an emerging method termed m^6^A-seq or MeRIP-seq has received considerable attention recently due to its accuracy and reproducibility. This novel method is based on the high specificity of one antibody raised against m^6^A in combination of high-throughput sequencing, making the mapping of m^6^A in the mammalian transcriptome possible [23, 35].

However, the immunoprecipitation-based approach m^6^A-seq localizes m^6^A residues to 100- to 200-nt-long regions of transcripts and is incapable of single-nucleotide-resolution detection of methylation sites. Contrary to other base modifications, such as 5-methylcytosine (5^m^C), which can make use of chemical conversion of cytosine as a mapping approach at single-nucleotide resolution in RNA [36], m^6^A RNA methylation has no such chemical conversion. Additionally, m^6^A does not introduce errors during reverse transcription that would allow direct mapping of its position [37]. A novel method called m^6^A individual-nucleotide-resolution crosslinking and immunoprecipitation (miCLIP) has marked a major step forward in the field, which augments m^6^A-seq with UV-induced crosslinking of the antibody to the immunoprecipitated RNA fragments [38]. Reverse transcription of crosslinked RNA then results in a highly specific pattern of mutations or truncations in the cDNA, and these specific mutational signatures are crucial for mapping m^6^A residues throughout the transcriptome at single-nucleotide resolution.

Moreover, it is currently possible to directly test the influence of altering any modification site in many organisms with the use of CRISPR-based genome engineering. CRISPR multiplexing strategies could potentially permit interrogation of many sites in parallel and hasten functional discoveries and would be valuable for the research of m^6^A RNA methylation as a complementary approach [39].

## Multiple functions of m^6^A RNA methylation in cancer

**Fig. 2:**
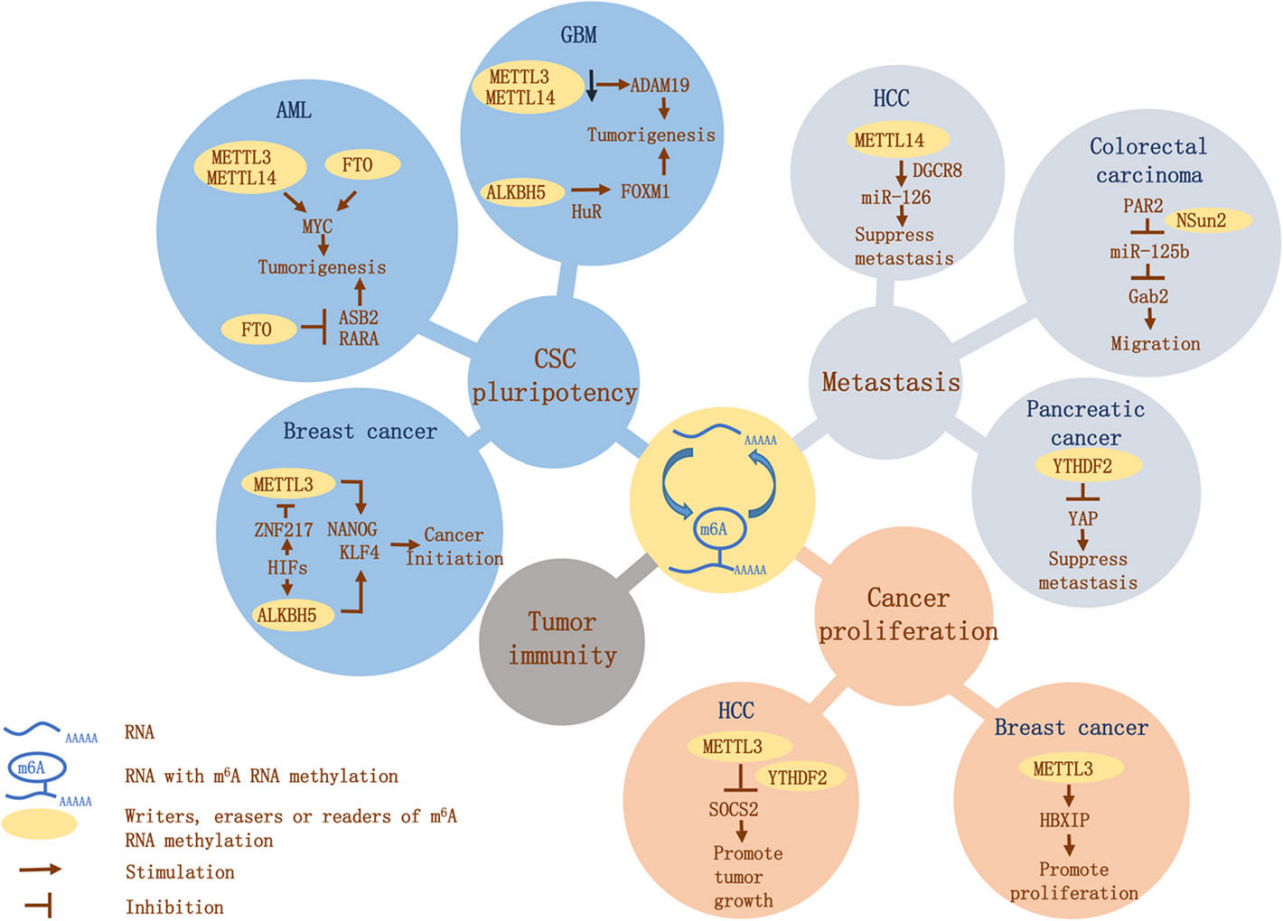
Multiple functions of m^6^A RNA methylation in cancer. m^6^A RNA methylation exerts multiple functions in cancer initiation and progression. The circle in the middle represents the reversible process of m6A RNA methylation, which plays a pivotal role in modulating CSC pluripotency, cancer proliferation, cancer metastasis, and tumor immunity. Distinct mechanisms through which m^6^A RNA methylation exerts these four aspects of functions in various types of cancers are shown in a graphical form in this picture

Currently, m^6^A RNA methylation has been found to have diverse biological regulatory functions in cancer initiation and progression and dysregulated expression of m^6^A RNA methylation is closely associated with various kinds of cancers. Herein, we mainly discuss four aspects of these functions exerted by m^6^A RNA methylation in cancer (Table 1, Fig. 2).

**Table 1:** Multiple functions exerted by m^6^A RNA methylation in various cancers

Molecule	Role in cancer	Cancer	Biological function	Mechanism	Refs
METTL3	Oncogene	AML	Promote tumorigenesis	Promote MYC et al. translation	[43]
	Oncogene	BC	Promote tumor growth	Promote HBXIP translation	[68]
	Oncogene	HCC	Promote tumor growth	Promote SOCS2 degradation	[70]
	Suppressor gene	GBM	Suppress tumorigenesis	Downregulate ADAM19	[58]
METTL14	Oncogene	AML	Promote tumorigenesis	Stabilize MYC and MYB	[44]
	Suppressor gene	HCC	Suppress metastasis	Promote miR126 processing	[71]
	Suppressor gene	GBM	Suppress tumorigenesis	Downregulate ADAM19	[58]
NSun2	Oncogene	CRC	Promote migration	Suppress miR125b processing	[73]
FTO	Oncogene	AML	Promote tumorigenesis	Destabilize ASB2 and RARA	[52]
	Oncogene	AML	Promote tumorigenesis	Stabilize MYC and CEBPA	[47]
ALKBH5	Oncogene	GBM	Promote tumorigenesis	Stabilize FOXM1 mRNA	[61]
	Oncogene	BC	Promote tumorigenesis	Stabilize NANOG and KLF4	[57]
YTHDF2	Oncogene	PC	Promote tumor growth	Activate Akt/GSK3b/CyclinD1	[75]
	Suppressor gene		Suppress metastasis	Destabilize YAP mRNAs	[75]

*GBM* glioblastoma, *CRC* colorectal carcinoma, *PC* pancreatic cancer

### m^6^A RNA methylation influences cancer stem cell pluripotency and cell differentiation

In recent years, a new dimension of intratumor heterogeneity and a hitherto-unappreciated subclass of neoplastic cells within tumors, termed cancer stem cells (CSCs), have aroused interests of researchers [40]. CSCs, typically rare within tumors, may prove to regenerate all facets of a tumor as a result of their stem cell-like capacity to self-renew, survive, and become dormant in protective microenvironments, representing a reservoir of self-sustaining cells that give rise to many types of cancers [41, 42].

Hematopoietic stem cells (HSCs) have well-defined developmental trajectories, and it is plausible to monitor and quantify their differentiation, providing an ideal model system in which to explore differentiation states. Abnormal or blocked differentiation is a common feature of myeloid hematological malignancies.

According to the recent research conducted by Ly P Vu and colleagues, leukemia cells show an elevated abundance of METTL3 as compared to normal hematopoietic cells. Utilizing miCLIP coupled with ribosome profiling, it is elucidated that METTL3 augments m^6^A levels of its target genes including myelocytomatosis (MYC), B cell lymphoma 2 (BCL2), and phosphatase and tensin homolog (PTEN) genes in the human acute myeloid leukemia (AML) MOLM-13 cell line, thereby promoting the translation of these mRNAs. These data suggest that AML cells regulate their translational state through m^6^A RNA methylation of specific transcripts to retain pluripotency properties and inhibit cell differentiation. Depletion of METTL3 in human myeloid leukemia cell lines induces cell differentiation and apoptosis and delays leukemia progression in recipient mice in vivo [43].

Consistent with METTL3, METTL14 is also highly expressed in AML cells carrying t(11q23), t(15;17), or t(8;21) and is downregulated during myeloid differentiation. Mechanistically, METTL14 exerts its oncogenic role by regulating its mRNA targets (e.g., myelocytomatosis (MYB) and MYC) through m^6^A modification, while the protein itself is negatively regulated by spleen focus-forming virus proviral integration oncogene (SPI1), collectively forming a SPI1/METTL14/MYB, MYC, et al. signaling axis in myelopoiesis and leukemogenesis. This highlights the critical roles of METTL14 and m^6^A modification in normal and malignant hematopoiesis [44].

RBM15, another component of the m^6^A writer complex, is linked to myeloid leukemia as well where AML initiation is mediated by a chromosomal translocation t (1;22) of RBM15 (also called OTT1) with the myelin and lymphocyte (MAL) gene [45]. Bansal and colleagues also suggested a role for m^6^A RNA methylation in myeloid leukemia that WTAP expression was elevated in cells derived from 32% of patients with AML and knockdown of WTAP resulted in reduced proliferation, increased differentiation, and increased apoptosis in a leukemia cell line [46].

However, upregulated m^6^A RNA methylation expression can result in anti-proliferation effects in some circumstances. Su and colleagues have elaborated that R-2-hydroxyglutarate (R-2HG) exhibits broad and variable anti-proliferation effects in leukemia and glioma since it increases global m^6^A RNA modification in the sensitive cells via suppressing FTO. The R-2HG/FTO/m^6^A axis regulates MYC and CCAAT/enhancer-binding protein alpha (CEBPA) gene expression and downstream pathways [47].

On the other hand, decreased m^6^A RNA methylation levels may exert oncogenic functions in some certain types of AML. For example, FTO, an m^6^A demethylase, is highly expressed in AML with t(11q23)/MLL rearrangements, t(15;17)/PML-RARA, FLT3-ITD, and/or NPM1 mutations and functions as an oncogene that promotes leukemic oncogene-mediated cell transformation and inhibits all-trans-retinoic acid (ATRA)-mediated leukemia cell differentiation. The oncogenic role of FTO is exerted through regulating expression of its target genes such as a suppressor of cytokine signaling box-2 (ASB2) and the retinoic acid receptor alpha (RARA) by reducing m^6^A RNA methylation levels in these mRNA transcripts. RNA stability assays have proposed that FTO-induced repression of ASB2 and RARA expression is at least in part due to the decreased stability of ASB2 and RARA mRNA transcripts upon FTO-mediated decrease of m^6^A RNA methylation levels. However, the reader(s) that targets m^6^A modification and impairs mRNA stability remains to be identified, which indicates an additional reading process controlling the stability of FTO target transcripts. Studies have [48–51] demonstrated the anti-leukemic effects of ASB2 and RARA, suggesting that the FTO/ASB2 or RARA axis likely plays a critical role in the pathogenesis of AML [52].

In breast cancer, Zhang and colleagues proposed that the exposure of breast cancer cells to hypoxia could stimulate hypoxia-inducible factor (HIF)-1α- and HIF-2α-dependent expression of ALKBH5, which induced m^6^A demethylation and stabilization of NANOG mRNA. Intratumoral hypoxia is a critical feature of the tumor microenvironment caused by dysregulated cell proliferation in combination with abnormal blood vessel formation and function, which drives cancer progression [53–55]. NANOG, as a pluripotency factor, is required for primary tumor formation and metastasis since they play a pivotal role in the maintenance and specification of cancer stem cells. This explains the correlation between decreased m^6^A RNA methylation level and promotion of breast cancer stem cell (BCSC) phenotype [56]. The researchers have also reported that the exposure of breast cancer cells to hypoxia induces zinc finger protein 217 (ZNF217)-dependent inhibition of m^6^A methylation of mRNAs encoding NANOG and Kruppel-like factor 4 (KLF4), which is another pluripotency factor that mediates BCSC speciation [57].

When it comes to glioblastoma (GBM), a study carried out by Cui et al. demonstrates that reduced mRNA m^6^A level is critical for maintaining glioblastoma stem-like cell (GSC) growth, self-renewal, and tumor development as downregulation of METTL3 or METTL14 expression reduces mRNA m^6^A levels of theirs target gene A disintegrin and metallopeptidase domain 19 (ADAM19) and promotes ADAM19 expression. ADAM19 is a metalloproteinase disintegrin gene that exhibits elevated expression in glioblastoma cells and promotes glioblastoma cell growth and invasiveness [58–60].

Similarly, Zhang and colleagues have identified that m^6^A demethylase ALKBH5 is highly expressed in GSCs and demethylates forkhead box protein M1 (FOXM1) nascent transcripts [61]. The nuclear RNA-binding protein HuR, which reportedly regulates both pre-mRNA splicing and expression [62, 63], has been shown to bind with RNAs with no m^6^A modification and exert stabilizing effects on its bound RNAs [24]. Recruiting HuR to the unmethylated 3′UTR makes FOXM1 nascent transcripts more stable and upregulates its expression. Accumulating evidence has shown that the transcription factor FOXM1 functions as a key cell-cycle molecule required for G1/S and G2/M transition and M-phase progression [64] and is overexpressed in GBM, playing a pivotal role in regulating GSC proliferation, self-renewal, and tumorigenicity [65–67]. Taken together, lower m^6^A level mediated by ALKBH5 in GBM helps to promote tumor progression.

### m^6^A RNA methylation involves in cancer cell proliferation

m6A RNA methylation has been found involved in cancer cell proliferation in many kinds of cancers. There remains a coherent natural link between the oncoprotein hepatitis B virus X-interacting protein (HBXIP) and METTL3 in the development of breast cancer (BC). Mechanistically, HBXIP upregulates METTL3 in breast cancer cells by inhibiting miRNA let-7g, which downregulates the expression of METTL3 by targeting its 3′UTR. METTL3 then promotes the expression of HBXIP gene through m^6^A modification, forming a positive feedback loop of HBXIP/let-7g/METTL3/HBXIP, which leads to accelerated cell proliferation in breast cancer [68].

In human hepatocellular carcinoma (HCC), METTL3 has been elucidated to be frequently upregulated and contributes to HCC progression through a distinct mechanism. SOCS2, one of the members of suppressor of cytokine signaling (SOCS) family, functions as a tumor suppressor gene by negatively regulating the JAK/STAT pathway [69]. METTL3 substantially augments SOCS2 mRNA m^6^A modification and downregulates SOCS2 mRNA expression by degrading SOCS2 mRNA transcripts through m^6^A “reader” protein YTHDF2-dependent degradation pathway, suggesting a new dimension of epigenetic alteration in liver carcinogenesis [70].

### m^6^A RNA methylation promotes cancer cell migration and tumor metastasis

The topic that m^6^A RNA methylation can act on cancer cell migration and tumor metastasis has now blossomed into a full-fledged field of research.

In HCC, especially in metastatic HCC, a decreased tendency of m^6^A modifications is observed and METTL14 is addressed to be the main factor involved in aberrant m^6^A modification [71]. It has been demonstrated by Alarcon and colleagues that m^6^A modification can mark pri-miRNAs for processing by recognizing DiGeorge critical region 8 (DGCR8) in a manner dependent on METTL3/m^6^A, highlighting the important role of m^6^A modification in RNA processing, including mRNAs and pri-miRNAs [32]. Similarly, METTL14 manipulates pri-miRNA processing by regulating the recognition and binding of DGCR8 to pri-miRNAs in an m^6^A-dependent manner and thus METTL14 depletion in HCC results in pri-miR126 processing arrest and reduces the expression of mature miR126 who has been identified as a metastasis suppressor, leading to advanced metastasis capability.

Besides the genetic background of cancer cells, alteration in microenvironment has emerged as a vital layer of regulating cancer metastasis. In colorectal carcinoma (CRC), serine proteases are important component in microenvironment and can selectively activate protease-activated receptor 2 (PAR2) through proteolysis of the receptor [72]. The research conducted by Yang and colleagues has unraveled that PAR2 activation decreases the level of miR-125b through NOP2/Sun RNA methyltransferase family, member 2 (NSun2)-mediated pre-miR-125b2 methylation in CRC. NSun2 is discovered to methylate precursor of miR-125b, interferes with its processing, and reduces the level of mature miR-125b [73]. The downregulation of miR-125b augments the expression of its target gene GRB2-associated-binding protein 2 (Gab2), thereby dramatically promoting cancer cell migration, which provides a novel epigenetic mechanism by which m^6^A modification on miRNAs promotes cancer metastasis [74].

In human pancreatic cancer (PC), the research conducted by Chen et al. showed that YTHDF2 was upregulated at both mRNA and protein levels and orchestrated proliferation and epithelial–mesenchymal transition (EMT) dichotomy [75]. Two of the main characteristics of tumor growth are uncontrolled proliferation and abnormal cell migration [76, 77]. However, cells are usually not supposed to respond to gene alterations by proliferating or migrating both at the same time, which is called migration–proliferation dichotomy [78]. YTHDF2 functions as the main regulator in this phenomenon who can promote the ability of proliferation via Akt/GSK3b/CyclinD1 pathway, while it can suppress the migration, invasion, and adhesion ability by inhibiting EMT probably via downregulation of yes-associated protein (YAP) gene. There exist two m^6^A RNA methylation sites in YAP mRNA transcript with one site in coding DNA sequences (CDS) and the other site in exon [35]. Therefore, it seems reasonable to come to the hypothesis that YTHDF2 might bind to m^6^A sites of YAP mRNA to decrease the stability of mRNA, while the direct link between YTHDF2 and YAP remains to be clarified.

### m^6^A RNA methylation may contribute to tumor immunity

As elucidated by Li et al., m^6^A mRNA methylation controls T cell homeostasis by targeting the IL-7/STAT5/SOCS pathway. This prompts the very first attempt at studying m^6^A RNA methylation as key regulators of T cell differentiation, suggesting their involvement in tumor–immune system communication and importance in tumor growth and spread [79].

In other circumstances, toll-like receptors (TLRs) establish the first line of defense against besieging pathogens as the most conserved molecules of the innate immune system, which recognize pathogen-associated molecular patterns to facilitate an immune response. RNAs with m^6^A modification are found incapable of activating TLR3, and those with 5^m^C and/or m^6^A do not activate TLR7 or TLR8, leading to non-recognition of pathogens carrying these RNA modifications by TLR receptors (e.g., a viral nucleic acid). The methylation in m^6^A interferes with Watson–Crick base pairing; thus, its presence destabilizes RNA duplexes, which may explain why RNAs containing m^6^A modification are incapable of stimulating TLR3 [80]. Dendritic cells (DCs) exposed to such modified RNA express significantly less cytokines and activation markers than those treated with unmodified RNA. These undetected viral components may then stimulate a pathway involved in cancer development [81–83].

## The future of m^6^A RNA methylation

### m^6^A RNA methylation as diagnostic and therapeutic targets

Huang et al. have demonstrated a significant increase of m^6^A RNA methylation in circulating tumor cells (CTCs) compared with whole blood cells for the first time, constituting the first step for the investigation of RNA methylation in CTCs from lung cancer patients. This research outcome may facilitate uncovering the metastasis mechanism of cancers in the future. It is worth noting that early detection and characterization of upregulated m^6^A expression in CTCs can make contributions to monitoring and preventing the development of overt metastatic diseases [84].

Moreover, m^6^A RNA methylation represents brand new therapeutic targets. All the subtypes of AMLs with high levels of endogenous FTO expression, such as those carrying t(11q23), t(15;17), NPM1 mutations, and/or FLT3-ITD, are more sensitive to ATRA treatment than the other AML subtypes [52]. It is plausible to hypothesize that the proliferation of these subtypes of AML cells relies more on the FTO signaling, and thus they are more responsive to ATRA treatment, as ATRA can release the expression/function of ASB2 and RARA, two negative targets of FTO, and thereby trigger cell differentiation.

Meclofenamic acid (MA) is a US Food and Drug Administration (FDA)-approved non-steroidal anti-inflammatory drug primarily known for its inhibition of prostaglandins synthesis as well as for its more specific inhibition of cyclooxygenase enzymes and lipoxygenases [85]. Intriguingly, recently identified FTO inhibitors are also derived from dual cyclooxygenase/lipoxygenase inhibitors [86]. Mechanistic studies conducted by Huang et al. indicated that MA specifically competed with FTO binding for the m^6^A-containing nucleic acid and treatment of HeLa cells with the ethyl ester form of MA (MA2) led to elevated levels of m^6^A modification in mRNA [87]. The identification of MA as a highly selective inhibitor of FTO will certainly shed light on more research into specific inhibitors existing within our resources of known drugs. Studies of MA2 have presented possibilities for therapeutic targets in cancers. For example, as elucidated by Cui and colleagues, MA2 suppresses glioblastoma progression and prolongs lifespan of GSC-grafted animals, which suggests that m^6^A methylation could be a promising target for anti-glioblastoma therapy [58].

The cellular components of m^6^A regulatory complex themselves can also function as novel options for cancer treatment. For example, researchers have endeavored to design a peptide mimicking the critical METTL14 domain and investigate whether it has a potential therapeutic effect in HCC [71].

However, the correlation between m^6^A RNA methylation and clinical features including predict treatment-free survival (TFS) and overall survival (OS) in cancer still warrants further research.

### Challenges of future research on m^6^A RNA methylation

As described above, the rapid development of m^6^A RNA methylation research has contributed to revealing underlying mechanisms of cancer initiation and progression. However, there remain a large number of challenges.

The first issue we should pay attention to is that whether the multiple functions proposed in cancer is actually dependent on m^6^A modification. Cellular components of m^6^A methylation complex are well established to be involved in cancer carcinogenesis and may function as tumor promoters or suppressors [56, 88–93]. It is identified by Lin and colleagues that METTL3 boosts translation of certain mRNAs including epidermal growth factor receptor (EGFR) and the Hippo pathway effector TAZ, thus promoting growth, survival, and invasion of human lung cancer cells. METTL3 activates mRNA translation through an interaction with translation initiation machinery rather than invoking m^6^A reader proteins downstream of nuclear METTL3, since both wild-type and catalytically inactive METTL3 promote translation when tethered to a reporter mRNA [56].

Additionally, the elevated level of the S-adenosyl methionine (SAM) donor of the methyl group in the m^6^A methylation process has been shown to suppress cell growth in cancer [94–98]. However, a direct causative link between m^6^A RNA methylation caused by SAM and its cancer growth-inhibitory effect remains to be established.

Secondly, we need to identify through which mechanism m^6^A RNA methylation is introduced. Despite the strong consensus, only a small fraction of RACH sites is detectably methylated in vivo, arguing that the sequence motif is not sufficient to determine the distribution of m^6^A [39]. It has been elucidated by Zhou and colleagues that miRNAs regulate m^6^A modification via a sequence pairing mechanism, through which miRNA sequences alter m^6^A modification levels via modulating the binding of METTL3 to mRNAs containing miRNA targeting sites. These results reveal how miRNAs involves in regulating the formation of m^6^A and partially explain the site selection mechanism of m^6^A [99]. Similarly, as reported by Zhang et al., the interaction between ALKBH5 and FOXM1 nascent transcripts is facilitated by a nuclear antisense lncRNA FOXM1-AS [61]. More than 70% of mammalian transcriptomes have antisense transcription [100]. It is worthwhile to determine whether there are other antisense lncRNAs that give rise to the specificity of m^6^A methylation so that the current view of methylation regulation can be completed.

Another mechanism by which the emplacement of m^6^A is regulated refers to that METTL3 can associate with chromatin and localize to the transcriptional start sites of active genes. Thereby, METTL3 induces m^6^A modification within the coding region of the associated mRNA transcript and enhances its translation by relieving ribosome stalling. One example is that METTL3 modulates nn n zSP1, an oncogene in several cancers which regulates c-MYC expression in this way [101], identifying a new paradigm for selecting RNAs to be modified, namely the stable recruitment of the RNA-modifying enzyme to specific genomic loci [102].

Iron metabolism also participates in m^6^A recruitment modulation. ALKBH5 belongs to the AlkB family of non-heme Fe(II)/a-ketoglutarate-dependent dioxygenases, whose activity is iron dependent [17]. Gene expression profiling carried out by Schonberg et al. revealed ferritin-dependent regulation of FOXM1 signaling. Given that Zhang et al. have proposed the interaction between ALKBH5 and FOXM1 nascent transcripts, it is plausible to indicate that ALKBH5 activity can be modulated by iron metabolism, consequently affecting FOXM1 expression [61, 66].

The third issue is that although there exist emerging links between m^6^A RNA methylation and transcript-specific fates in different cell types, it remains to be determined whether m^6^A is differentially recognized by distinct YTH domain proteins or whether m^6^A RNA methylation is used in a cell type-specific manner to either direct translational enhancement or mRNA decay.

Interestingly, METTL14 promotes stability of MYC mRNA through affecting m^6^A abundance mainly on the 3′-terminal exon, while FTO promotes stability of MYC mRNA through inhibition of YTHDF2-mediated RNA decay which can be attributed to decreased m^6^A abundance on the 5′-terminal and internal exons of MYC mRNA [52]. This phenomenon indicates that m^6^A modifications on different regions of the same mRNA transcript (e.g., MYC) may result in distinct fates likely due to recognition by different readers. It also leads to very open and constructive discussion that YTH domain family proteins may depend on sequence context to control their selectivity towards m^6^A sites and may be dynamically regulated.

However, much remains to be learned before we can understand the dynamic regulation of the co-transcriptional installation of m^6^A RNA methylation and its impacts on downstream mRNA processing events and cancer progression.

## Conclusion

As the most prevalent modifications of mRNA, m^6^A RNA methylation has a deep root in modulating gene expression and is implicated in affecting many cellular processes and diseases, including cancers. Over the past few years, empowered by the availability of highly specific antibodies and the accessibility of high-throughput sequencing technologies, it has become feasible to identify the chemical basis and multiple functions of m^6^A RNA methylation. There remain many other kinds of RNA post-transcriptional modifications which may share similar properties with m^6^A RNA methylation, shedding light on the advancement of epitranscriptome.

## Abbreviations

5^m^C: 5-Methylcytosine
ADAM19: A disintegrin and metallopeptidase domain 19
ALKBH5: alkB homolog 5
AML: Acute myeloid leukemia
ASB2: A suppressor of cytokine signaling box-2
ATRA: All-trans-retinoic acid
BC: Breast cancer
BCL2: B cell lymphoma 2
BCSC: Breast cancer stem cell
CDS: Coding DNA sequences
CEBPA: CCAAT/enhancer-binding protein alpha
CRC: Colorectal carcinoma
CSCs: Cancer stem cells
CTCs: Circulating tumor cells
DC: Dendritic cells
DGCR8: DiGeorge Critical Region 8
DNMT: DNA methyltransferases
EGFR: Epidermal growth factor receptor
EMT: Epithelial–mesenchymal transition
FDA: Food and Drug Administration
FOXM1: Forkhead box protein M1
FTO: Fat mass and obesity-associated protein
Gab2: GRB2-associated-binding protein 2
GBM: Glioblastoma
GSCs: Glioblastoma stem-like cells
HBXIP: Hepatitis B virus X-interacting protein
HCC: Hepatocellular carcinoma
HIF: Hypoxia-inducible factor
HSCs: Hematopoietic stem cells
KLF4: Kruppel-like factor 4
LC–MS/MS: Liquid chromatography coupled to triple–quadrupole mass spectrometry
m^6^A: *N*^6^-Methyladenosine
MA: Meclofenamic acid
MA2: The ethyl ester form of MA
MAL: Myelin and lymphocyte
METTL: Methyltransferase-like
miCLIP: m6A individual-nucleotide-resolution crosslinking and immunoprecipitation
MYB: Myeloblastosis
MYC: Myelocytomatosis
NSun2: NOP2/Sun RNA methyltransferase family, member 2
OS: Overall survival
PAR2: Protease-activated receptor 2
PC: Pancreatic cancer
PTEN: Phosphatase and tensin homolog
R-2HG: R-2-hydroxyglutarate
RARA: Retinoic acid receptor alpha
RBM15: RNA-binding motif protein 15
SAM: S-Adenosyl methionine
SOCS: Suppressor of cytokine signaling
SPI1: Spleen focus-forming virus proviral integration oncogene
SRSF: Serine and arginine-rich splicing factor
TFS: Treatment-free survival
TLRs: toll-like receptors
WTAP: Wilms tumor 1-associated protein
YAP: yes-associated protein
YTH: YT521-B homology
ZNF217: Zinc finger protein 217
